# Creutzfeldt–Jakob Disease Presenting With Dizziness and Gaze-Evoked Nystagmus: A Case Report

**DOI:** 10.1097/MD.0000000000002766

**Published:** 2016-02-18

**Authors:** Yun-Ju Choi, Kyung-Wook Kang, Sae-Young Lee, Seung-Ho Kang, Seung-Han Lee, Byeong C. Kim

**Affiliations:** From the Department of Neurology, Chonnam National University Medical School, Gwangju (Y-JC, K-WK, S-YL, S-HK, S-HL, BCK); and Department of Neurology, Presbyterian Medical Center (Y-JC), Jeonju, Korea.

## Abstract

Sporadic Creutzfeldt–Jakob disease (CJD) is clinically characterized by rapidly progressive dementia combined with other cardinal symptoms, such as myoclonus, visual or cerebellar disturbances, extrapyramidal or pyramidal disturbance, and akinetic mutism. However, as an initial manifestation, focal neurologic deficits other than the aforementioned or nonspecific generalized symptoms may lead to a misdiagnosis or a delayed diagnosis.

The authors report a case of 66-year-old male patient with sporadic CJD who had dizziness, gaze-evoked nystagmus (GEN), and other central eye signs (impaired smooth pursuit, saccadic dysmetria) as an initial manifestation without dementia. The central eye signs led us to perform brain magnetic resonance images, which showed abnormal cortical high-signal intensity in both the cerebral and cerebellar hemispheres including the vestibulocerebellum. We reached a presumptive diagnosis of CJD, but the findings did not meet diagnostic criteria for probable CJD at that time. Three weeks after the initial work-ups, the patient presented with typical neurological findings of CJD: rapidly progressive dementia, akinetic mutism, and myoclonus of the left arm. Cerebrospinal fluid was positive for 14-3-3 protein, and electroencephalography showed periodic sharp wave complexes.

In this patient, GEN and other central eye signs provided diagnostic clues for CJD. These unusual neurological manifestations may help physicians have a thorough knowledge of early deficits of CJD.

## Abstract 2

Supplemental Digital Content is available in the text

## INTRODUCTION

Sporadic Creutzfeldt–Jakob disease (CJD) is a fatal neurodegenerative disease, which is characterized by rapidly progressive dementia and a short duration of illness.^1^ Along with progressive dementia, most patients present with a variety of neurological symptoms, including myoclonus, cerebellar or visual disturbances, extrapyramidal or pyramidal disturbance, and akinetic mutism.^1^ In some cases, however, focal neurological deficits other than the aforementioned or nonspecific generalized symptoms could be manifested as an initial symptom,^2^ which may lead to a misdiagnosis or a delayed diagnosis of CJD. To the best of our knowledge, dizziness and oscillopsia with nystagmus have rarely been shown to be initial symptoms of CJD. We describe a patient with sporadic CJD who initially had dizziness for several weeks, and then gaze-evoked nystagmus (GEN) and other central eye signs pointing to a cerebellar lesion before a diagnosis of clinically probable CJD was made.

## CASE REPORT

A 66-year-old man, with no remarkable past medical history, visited our hospital due to aggravating dizziness and oscillopsia for 4 weeks. At that time, he visited a private clinic with the symptoms, and the diagnosis was peripheral vestibulopathy.

**FIGURE 1:**
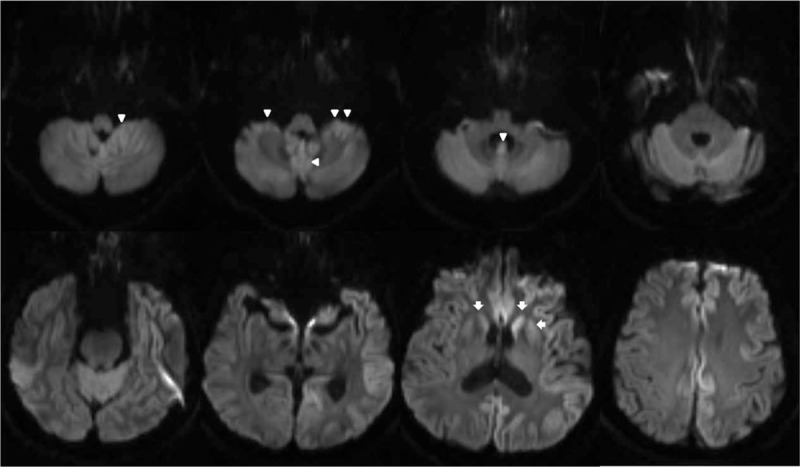
Diffusion-weighted images show high-signal intensity in the cerebellar cortex, especially in the nodulus and anterior cerebellum including the flocculus (arrow heads), as well as the fronto-temporo-occipital cortex and basal ganglia (arrows) with a left-sided predominance.

On admission, the neurological examination revealed GEN on eccentric gaze as well as rebound nystagmus on resuming the straight-ahead position (video SDC). Also, the patient showed mild cerebellar dysfunctions, including limb and gait ataxia. However, motor and sensory functions were normal. He did not show apraxia, acalculia, aphasia, or other higher cortical (frontal executive and visuospatial) dysfunctions. The mini-mental state examination result was 29/30 points. Video-oculography exhibited GEN, impaired smooth pursuit, and saccadic dysmetria with normal velocity and latency. Because the bedside and laboratory eye signs pointed to central pathology, a lesion of the vestibulocerebellum, we continued thorough diagnostic procedures to find the causative lesion. Brain magnetic resonance (MR) images were obtained, and the diffusion weighted image (DWI) showed abnormal cortical high signal intensity in both the cerebral and cerebellar hemispheres including the vestibulocerebellum (Figure 1). The initial electroencephalography (EEG) showed slow wave activity in the delta/theta range with a frontal predominance. We reached a presumptive diagnosis of CJD after DWI, but the findings did not meet diagnostic criteria for probable CJD at that time.

Three weeks after the initial workup, the patient presented with typical neurological findings of CJD: rapidly progressive dementia, akinetic mutism, and myoclonus of the left arm. The mini-mental state examination result was 22/30 points, and the follow-up test result obtained 1 week later was 12/30. Cerebrospinal fluid (CSF) was positive for 14-3-3 protein, and follow-up EEG showed periodic sharp wave complexes (PSWCs). With the clinical (at least 2 out of 4: dementia, cerebellar or visual, pyramidal or extrapyramidal, and akinetic mutism) and laboratory findings (at least 1 out of 3: PSWCs on EEG, 14-3-3 detection in CSF, and high-signal abnormalities in the caudate nucleus and putamen; or at least 2 cortical regions on either DWI or FLAIR), our patient was finally diagnosed with probable CJD.^1^ There was neither known familial history of prion diseases nor iatrogenic contamination. Also, prion protein (PRNP) gene mutation was not observed. We concluded that the patient had a sporadic type of CJD.

After the diagnosis of probable CJD, the patient was managed according to the guidelines of the Korea Centers for Disease Control and Prevention. Although transmission of CJD is very rare in a clinical setting, the potential for transmission by means of contaminated instruments or higher-infectivity tissues should be eliminated.^3^ During the follow-up, he had complications, such as aspiration pneumonia and urinary tract infection, not to mention cardinal symptoms of CJD. Thus, the patient received symptomatic and supportive treatment. Despite the meticulous treatment, the patient died of pneumonia 9 months after the disease onset.

Informed consent was obtained after the nature and possible consequences of this case report were explained to the patient and his caregivers.

## DISCUSSION

Established clinical diagnostic criteria for sporadic CJD rely primarily on neurological symptoms that may appear late in the disease course. At least 2 of the 4 cardinal symptoms should be accompanied by progressive dementia to establish a diagnosis of CJD.^1^ However, initial symptoms may be nonspecific (ie, lightheadedness, anxiety, sleep habit change, weight loss, and frequent falls), and various focal neurological signs (ie, aphasia, vertical gaze palsy, homonymous hemianopsia, and auditory agnosia) have been reported.^2^ In a previous report, ∼80% of all sporadic CJD patients show focal signs in the early stage,^4^ so an initial diagnosis could be challenging.

Dizziness/vertigo presentation is infrequent in CJD. A meta-analysis reported that dizziness/vertigo is present as an initial manifestation in 2.6% of all CJD patients.^5^ Furthermore, as the initial manifestation of CJD can closely mimic benign peripheral vestibulopathy, such as vestibular neuritis and labyrinthitis, without definite cerebellar dysfunction and other central signs,^2,6^ it may lead to a delayed diagnosis or a misdiagnosis. Cerebellar dysfunction is common in CJD throughout the illness (42–86%); however, it occurs as an initial manifestation only in ∼20% of patients.^5^

During a detailed neurological examination, we found central types of GEN and rebound nystagmus due to the impairment of gaze holding caused by lesions in the cerebellum including the flocculus and paraflocculus. We performed further diagnostic workup using brain MR imaging on the basis of the central eye signs, which gave a useful diagnostic clue to CJD. Besides nystagmus, smooth pursuit abnormalities and saccadic dysmetria would be other cerebellar eye signs. In our patient, cerebellar lesions on DWI may have led to clinical symptoms and signs.

Abnormal eye movements have been reported in some patients with an ataxic-cerebellar form of CJD. There is a report on periodic alternating nystagmus and slow vertical saccade occurring early in the course of CJD.^7^ Patients presenting with upbeat and GEN eventually show ocular dipping.^8^ These abnormal eye findings point to central pathology involving the vestibulocerebellum and brainstem, and would be a useful diagnostic clue especially to an ataxic-cerebellar form of CJD.

Here, we reported the case of a patient with sporadic CJD who had dizziness as an initial manifestation and met diagnostic criteria for probable CJD several weeks after symptom onset. In our patient, GEN and other central eye signs gave an important diagnostic clue to CJD. These unusual neurological manifestations may help clinicians have a thorough knowledge of early deficits of CJD.

## Supplementary Material

Supplemental Digital Content

## References

[1] Zerr I Kallenberg K Summers DM Updated clinical diagnostic criteria for sporadic Creutzfeldt–Jakob disease. *Brain* 2009; 132:2659–2668. 19773352 10.1093/brain/awp191 PMC2759336

[2] Bigelow DC Eisen MD Yen DM Otolaryngological manifestations of Creutzfeldt–Jakob disease. *Arch Otolaryngol Head Neck Surg* 1998; 124:707–710. 9639484 10.1001/archotol.124.6.707

[3] Rutala WA Weber DJ Creutzfeldt–Jakob disease: recommendations for disinfection and sterilization. *Clin Infect Dis* 2001; 32:1348–1356. 11303271 10.1086/319997

[4] Brown P Cathala F Castaigne P Creutzfeldt–Jakob disease: clinical analysis of a consecutive series of 230 neuropathologically verified cases. *Ann Neurol* 1986; 20:597–602. 3539001 10.1002/ana.410200507

[5] Appleby BS Appleby KK Rabins PV Does the presentation of Creutzfeldt–Jakob Disease vary by age or presumed etiology? A meta-analysis of the past 10 years. *J Neuropsychiatry Clin Neurosci* 2007; 19:428–435. 18070846 10.1176/jnp.2007.19.4.428

[6] Mantokoudis G Saber Tehrani AS Newman-Toker DE An unsual stroke-like clinical presentation of Creutzfeldt–Jakob disease: acute vestibular syndrome. *Neurologist* 2015; 19:96–98. 25888195 10.1097/NRL.0000000000000019

[7] Grant MP Cohen M Petersen RB Abnormal eye movements in Creutzfeldt–Jakob disease. *Ann Neurol* 1993; 34:192–197. 8338343 10.1002/ana.410340215

[8] Jeong SH Kim SY Park SH Ocular dipping in Creutzfeldt–Jakob disease. *J Neuroophthalmol* 2008; 28:293–295. 19145128 10.1097/WNO.0b013e31818e4023

